# Increased Ca^2+^ influx through Ca_V_1.2 drives aortic valve calcification

**DOI:** 10.1172/jci.insight.155569

**Published:** 2022-03-08

**Authors:** Maiko Matsui, Rihab Bouchareb, Mara Storto, Yasin Hussain, Andrew Gregg, Steven O. Marx, Geoffrey S. Pitt

**Affiliations:** 1 Cardiovascular Research Institute, Weill Cornell Medicine, New York, New York, USA.; 2 Zena and Michael A. Wiener Cardiovascular Institute, Icahn School of Medicine at Mount Sinai, New York, New York, USA.; 3 Division of Cardiology, Department of Medicine, and Department of Pharmacology, Vagelos College of Physicians and Surgeons, Columbia University, New York, New York, USA.

**Keywords:** Cardiology, Ion channels

## Abstract

Calcific aortic valve disease (CAVD) is heritable, as revealed by recent GWAS. While polymorphisms linked to increased expression of *CACNA1C* — encoding the Ca_V_1.2 L-type voltage-gated Ca^2+^ channel — and increased Ca^2+^ signaling are associated with CAVD, whether increased Ca^2+^ influx through the druggable Ca_V_1.2 causes CAVD is unknown. We confirmed the association between increased Ca_V_1.2 expression and CAVD in surgically removed aortic valves from patients. We extended our studies with a transgenic mouse model that mimics increased Ca_V_1.2 expression within aortic valve interstitial cells (VICs). In young mice maintained on normal chow, we observed dystrophic valve lesions that mimic changes found in presymptomatic CAVD and showed activation of chondrogenic and osteogenic transcriptional regulators within these valve lesions. Chronic administration of verapamil, a Ca_V_1.2 antagonist used clinically, slowed the progression of lesion development in vivo. Exploiting VIC cultures, we demonstrated that increased Ca^2+^ influx through Ca_V_1.2 drives signaling programs that lead to myofibroblast activation of VICs and upregulation of genes associated with aortic valve calcification. Our data support a causal role for Ca^2+^ influx through Ca_V_1.2 in CAVD and suggest that early treatment with Ca^2+^ channel blockers is an effective therapeutic strategy.

## Introduction

Calcific aortic valve disease (CAVD) is a life-threatening disorder affecting approximately 2% of people older than 65 years. Excess calcium phosphate deposition in the aortic valve stiffens the tissue and ultimately narrows the valve orifice. The consequent increase in left ventricular pressure strains the left ventricle. Common clinical outcomes, if this condition is untreated, are angina, heart failure, syncope, and/or sudden death. Surgical or transcatheter aortic valve replacement are the only current therapeutic options (1); no drug treatments have been shown to reverse or slow progression (2).

While the underlying mechanism of CAVD was previously thought to be wear and tear, researchers subsequently showed that calcification initiates after injury–mediated or inflammatory-mediated disruption of the valve endothelial surface, which leads to 2 different but overlapping responses, both of which start with differentiation of fibroblast-like valve interstitial cells (VICs) that reside below the endothelial surfaces (3). First, dystrophic calcification results from myofibroblast differentiation of VICs in response to paracrine action of TGF-β. Identifying features of VICs differentiated into myofibroblasts are expression of α-smooth muscle actin (α-SMA), which myofibroblasts organize into bundles or stress fibers, and that VICs become contractile. Activated myofibroblasts remodel the extracellular matrix that alters the local milieu in ways that promote calcification, such as by secretion of MMPs that release latent TGF-β within the extracellular matrix, thereby augmenting VIC differentiation to myofibroblasts. Activated myofibroblasts eventually undergo apoptosis and consequently deposit calcium salts that precipitate calcification (4). The second process by which VICs contribute to calcification is by undergoing osteogenesis. The ectopically transformed osteoblasts secrete bone matrix (5, 6), as reflected by upregulation of canonical osteogenic signaling pathways, such as the BMP pathway and the Wnt-signaling pathway, in diseased calcific aortic valves (6–8). Likewise, chondrogenic signaling in diseased valves is marked by the expression of *Aggrecan* (*ACAN*) and *SOX9*, a major chondrogenic transcriptional regulator (9).

Attempts at medical therapy to slow or ameliorate the development of CAVD have been unsuccessful, and failures have been attributed to the late initiation of treatment, when calcification is present and thus difficult to halt or reverse. Hence, while understanding the early events that eventually result in CAVD might provide opportunities to intervene in a timely manner, the initial events that activate VICs to cause dystrophic calcification or undergo osteogenesis are not well understood. Moreover, although the pathophysiological consequences of CAVD result primarily from stiffened valve leaflets, a recent study of donor heart valves from individuals not known to have aortic valve disease showed that nascent disease is characterized by intrinsic calcification at the hinge regions rather than in the leaflets (10). Focusing on these changes in the hinge regions, therefore, may offer a window of opportunity for therapeutic intervention.

One approach to identify the early changes is to focus on genetic susceptibility, because genetics represents factors continuously present and starting from birth. Indeed, GWAS have suggested genetic susceptibility to CAVD, but whether any of these associated loci are causal and the mechanisms by which variants increase susceptibility are largely unknown. An SNP near *LPA* demonstrated genome-wide significance (11), consistent with previous studies demonstrating an association between levels of the *LPA*-encoded lipoprotein(a) and aortic valve calcification (12–15). SNPs near *PALMD*, *IL6*, *ALPL*, and the noncoding *TEX41* also associate with aortic valve calcification (16–18), but whether variants at these loci are causal is unknown, as are the molecular mechanisms underlying disease association. A meta-analysis of 2 GWAS suggested an association between increased expression of *CACNA1C*, which encodes the Ca_V_1.2 L-type voltage gated Ca^2+^ channel, and CAVD (19). That association was supported by several additional observations, including increased *CACNA1C* transcripts in calcified versus noncalcified valves and a gene-set enrichment analysis of all SNPs (not only those within *CACNA1C*) with moderate (*P* < 0.0001) disease association that identified the calcium-signaling pathway (Kyoto Encyclopedia of Genes and Genomes identifier hsa04020) as the top gene set.

Ca_V_1.2 is the predominant voltage-gated Ca^2+^ channel in the myocardium, in which Ca_V_1.2 initiates excitation-contraction coupling. Additionally, Ca_V_1.2 serves essential roles in other excitable tissues, such as the brain and endocrine tissue, and in vascular smooth muscle cells, where it is the target of commonly prescribed Ca^2+^ channel blocker antihypertension drugs. Expression in cardiac valves had not been previously described, so a role in CAVD was unexpected. An increasing amount of evidence, however, suggests that Ca_V_1.2 exerts roles in nonexcitable tissues (20) and, as a plasma membrane voltage-gated Ca^2+^ channel, Ca_V_1.2 sits at the pinnacle of myriad intracellular Ca^2+^-dependent signaling events capable of triggering myofibroblast activation and osteogenic transformation. If increased Ca^2+^ influx through Ca_V_1.2 is causal for calcific aortic stenosis, then readily available FDA-approved drugs would be lead candidates for therapeutic intervention.

With human aortic valve samples, we confirmed increased Ca_V_1.2 in valves surgically excised for calcific aortic stenosis, compared with noncalcified valves. We then generated a mouse model to mimic increased Ca_V_1.2 expression within the aortic valve, with which we observed accelerated aortic valve calcification in vivo, thus showing that increased Ca_V_1.2 expression is, indeed, causal. Chronic administration of verapamil, a Ca_V_1.2 antagonist used clinically, slowed valve calcification, suggesting that reduction of Ca^2+^ influx through Ca_V_1.2 is a therapeutic opportunity. Using transcriptional profiling in cultured mouse VICs, we then defined how increased Ca^2+^ influx through Ca_V_1.2 could activate downstream signaling pathways capable of driving valve calcification.

## Results

**Figure 1:**
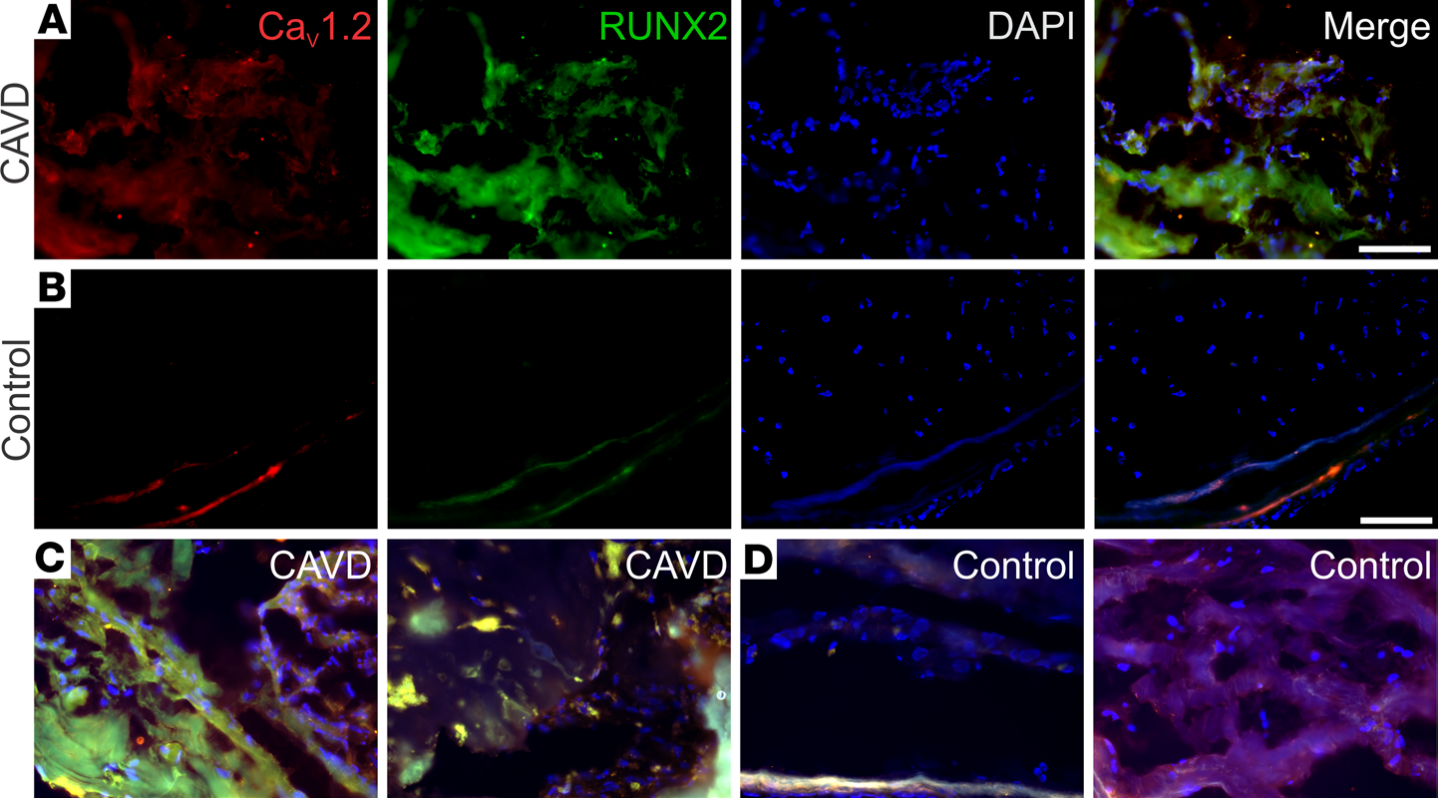
Increased Ca_V_1.2 and RUNX2 expression in calcified segments of aortic valves from individuals with CAVD. (**A** and **B**) IHC analysis of Ca_V_1.2 (red) and RUNX2 (green) of valve tissue excised from a patient with CAVD (**A**) or a valve excised from a heart harvested for transplant and without CAVD (**B**). The blue signal is DAPI, and merged images are shown in the right-most panels for each. (**C** and **D**) Merged images for 2 additional valves from patients with CAVD (**C**) and from valves excised from hearts harvested for transplant and without CAVD (**D**). Scale bars: 100 μm.

The researchers who conducted the GWAS that identified an association between *CACNA1C* and CAVD reported increased *CACNA1C* and *RUNX2* transcripts within calcified versus noncalcified human aortic valves (19). Using IHC, we found that calcified areas of valves obtained from individuals with CAVD valves also had increased levels of Ca_V_1.2 protein compared with valves obtained from individuals without CAVD (Figure 1A). Moreover, the increased signal for Ca_V_1.2 protein in valves obtained from individuals with CAVD was most prominent in areas that also displayed a strong signal for the osteogenic marker, RUNX2. In contrast, only a weak Ca_V_1.2 or RUNX2 signal was observed in valves excised from hearts harvested for transplant and without CAVD (Figure 1B). Three CAVD valves and 3 valves without CAVD were tested in toto with similar results (Figure 1, C and D).

**Figure 2:**
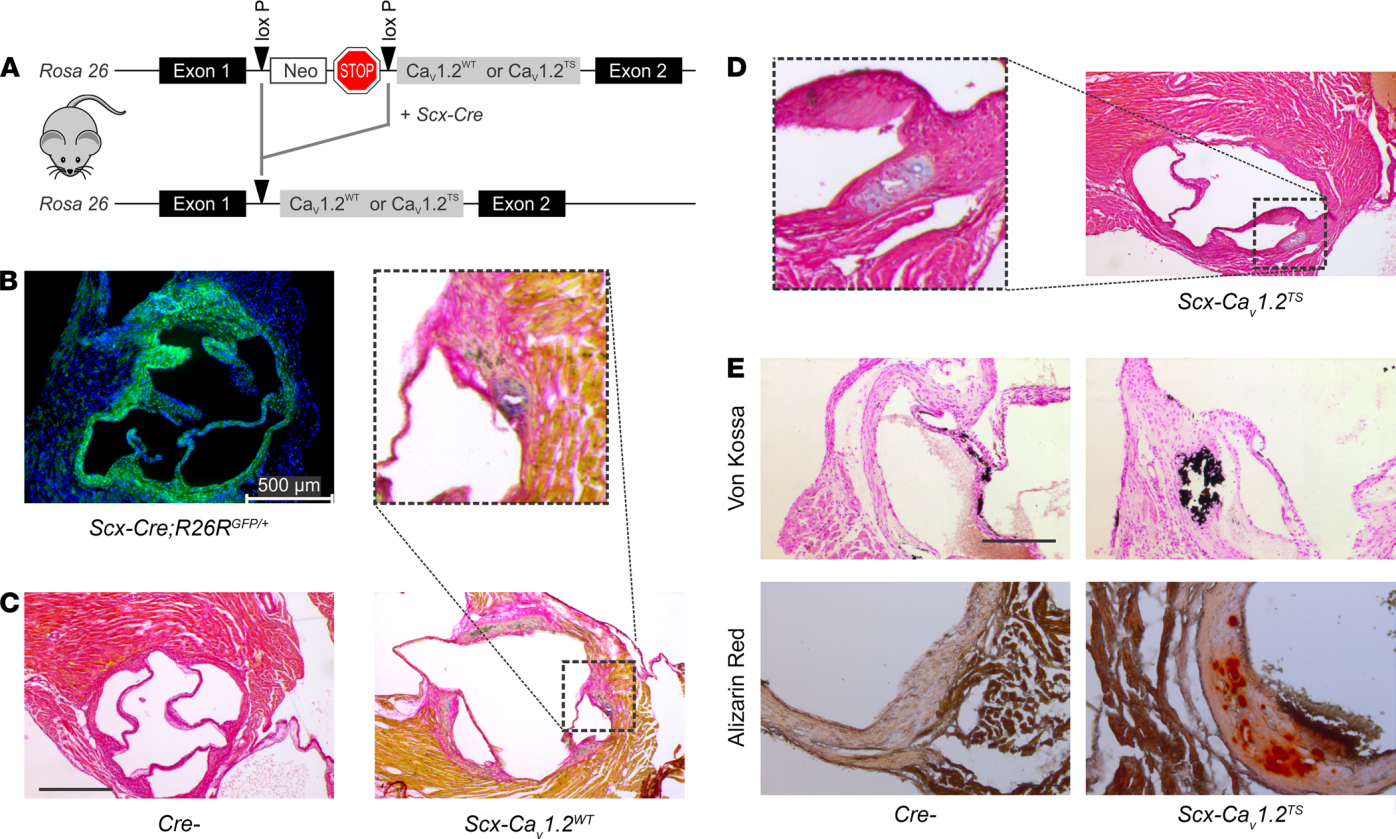
Increased Ca_V_1. 2 expression in the aortic valve is causal for CAVD. (**A**) Transgenic strategy for inducible Ca_V_1.2 expression. (**B**) GFP immunofluorescence (DAPI counterstain) showing aortic valve targeting with Scx-Cre. (**C** and **D**) Pentachrome staining shows lesions (inset) in Ca_V_1.2^WT^ (**C**) and Ca_V_1.2^TS^ (**D**) but not in Cre^–^ control mice (**C**). Scale bar: 200 μm. (**E**) Von Kossa and alizarin red stains demonstrate calcification of valve lesions in mice overexpressing Ca_V_1.2^TS^ in the valve. Scale bar: 100 μm.

To test if increased Ca_V_1.2 expression within the aortic valve is causal for valve calcification, we generated a mouse model in which transgenic Ca_V_1.2 expression was specifically increased in the valve tissue. We used a model in which transgenic Ca_V_1.2 expression was targeted specifically to the aortic valve, modifying a strategy we have used previously to target Ca_V_1.2 expression to the mandible (21) or osteoblasts (22, 23). Here, we crossed a *Scx-Cre* driver mouse with mice with a Ca_V_1.2 WT cDNA (Ca_V_1.2^WT^) inserted into *Rosa26* and downstream of a floxed STOP codon (Figure 2A). *Scx-Cre* drives expression in heart valves but not in the myocardium (24, 25), as we confirmed with a GFP reporter (Figure 2B). Thus, these *Scx-Ca_V_1.2^WT^* mice express transgenic Ca_V_1.2 specifically in the aortic valve. As we previously showed, this transgenic Ca_V_1.2 expression model increases Ca^2+^ influx (21) and thereby provides a model that mimics the increased Ca_V_1.2 transcription and protein levels observed specifically in the aortic valves of patients with CAVD (19) and the increased Ca_V_1.2 expression that we detected in a human aortic valve sample (Figure 1A).

Histological analyses with pentachrome staining of transgenic *Scx-Ca_V_1.2^WT^* valves obtained from 4-month-old mice showed the presence of discrete lesions in all valves examined (Figure 2C), but in only 1 valve from a Cre-negative control mouse (for Cre-negative control, *n* = 12; for *Scx-Ca_V_1.2^WT^*, *n* = 10; Fisher exact test, *P* < 0.0001). Thus, increased Ca_V_1.2^WT^ expression in the aortic valve was sufficient to cause development of calcified lesions. Also remarkable was that these lesions were present in 4-month-old mice, a stage much earlier than in typical murine CAVD models. Consistent with the relatively young age of the mice at which these lesions appeared, the lesions were predominantly located in the hinge regions rather than the valve leaflets, reflecting nascent disease seen in humans (10). Notably, the mice were maintained on a normal chow diet, in contrast to a high cholesterol diet commonly used in murine models of CAVD, and thus these early lesions do not require elevated lipid levels that are a CAVD risk factor.

We then developed a model to increase Ca^2+^ influx through Ca_V_1.2 to accelerate the disease process in the mouse model. We exploited a gain-of-function mutant G406R mutation (Ca_V_1.2^TS^) found in individuals with the multisystem disorder Timothy syndrome (26) that disrupts Ca_V_1.2 channel voltage-dependent inactivation and thereby promotes increased Ca^2+^ through the channel. Using the *Rosa26* knock-in strategy, but with the gain-of-function mutant Ca_V_1.2^TS^ expressed rather than the Ca_V_1.2^WT^ channel, we examined whether targeted expression in the aortic valve with *Scx-Cre* accelerated the appearance of calcified valve lesions. Figure 2D shows heterogeneous histological evidence of lesions in the valves from the *Scx-Ca_V_1.2^TS^* mice. We observed lesions in all (*n* = 22) *Scx-Ca_V_1.2^TS^* aortic valves examined but in only 1 valve from 12 Cre-negative control mice (Fisher exact test, *P* < 0.0001). Alizarin red and von Kossa staining demonstrated calcification within the lesions, but not in the adjacent histologically normal-appearing valve (Figure 2E), again consistent with the histological appearance and using the same detection methods as for the nascent lesions seen in humans (10). Thus, valve-specific expression of a gain-of-function mutant Ca_V_1.2^TS^ exacerbated the development of calcified lesions and provides a model to accelerate the development of aortic valve calcification.

**Figure 3:**
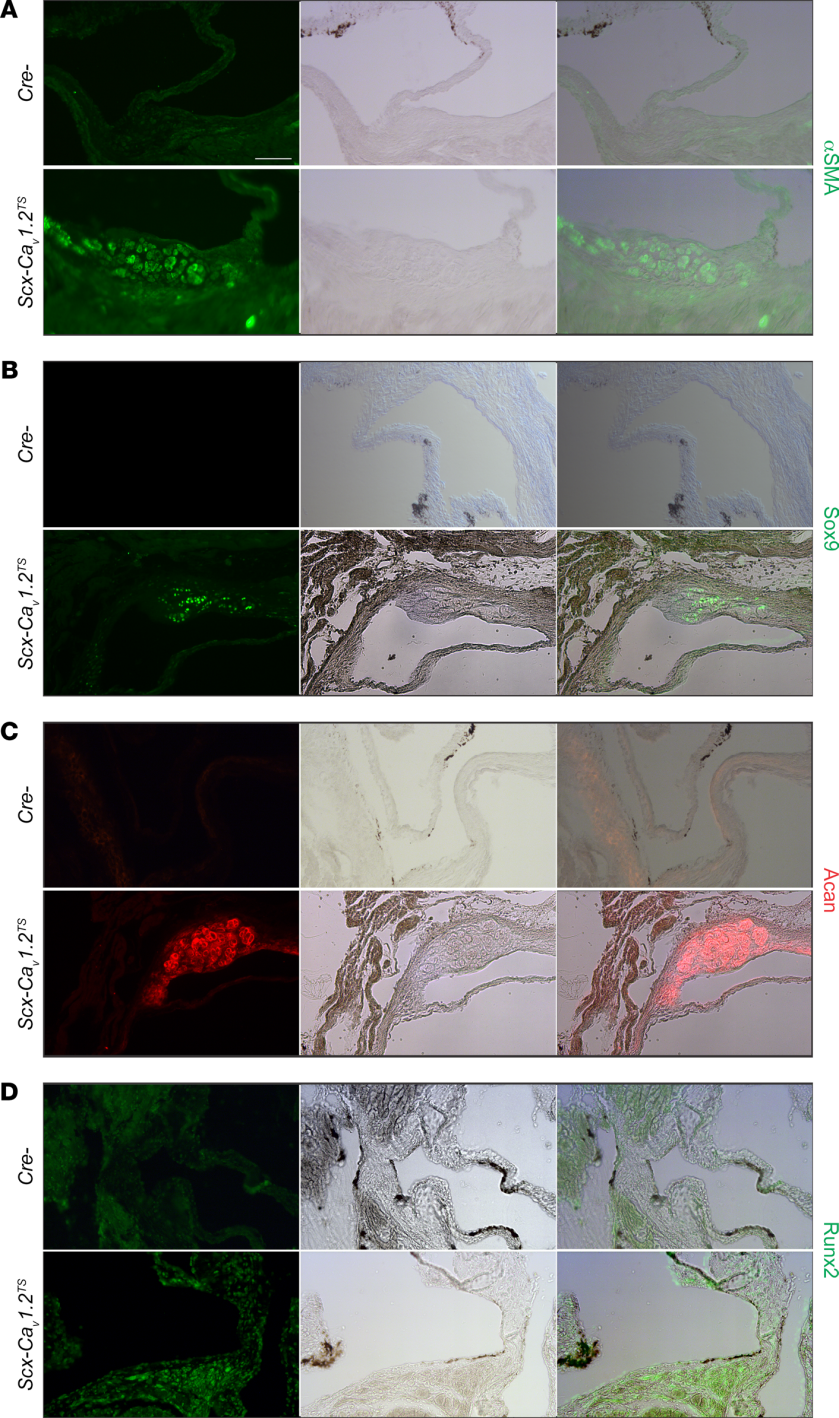
Lesion-specific upregulation of chondrogenic and osteogenic makers in vivo. Immunofluorescence for α-SMA (**A**), Sox9 (**B**), Aggrecan (**C**), and Runx2 (**D**) with accompanying light transmission and merged images of valves from *Cre^–^* control and *Scx-Ca_V_1.2^TS^* mice shows expression of the specific marker within the valve lesion but not in the unaffected valve. For each marker, examples from ≥3 samples. Scale bar: 100 μm.

Further characterization of the lesions with immunohistochemical markers revealed discrete dystrophic activation. For example, Figure 3A demonstrates increased α-SMA specifically within the discrete valve lesions in *Scx-Ca_V_1.2^TS^* (and not the *Cre^–^* control mice), but not in the unaffected adjacent valve tissue. Moreover, the lesions shown in Figure 3, B and C, display features of chondrogenesis: Sox9 and aggrecan (Acan) were both detected in the lesions in *Scx-Ca_V_1.2^TS^* mice but not in the unaffected valve areas. This finding is consistent with the recent discovery that *Sox9* and *Acan* are upregulated downstream of Ca^2+^ influx through Ca_V_1.2 in developing bone (27). Early features of osteogenesis, as indicated by the expression of the master osteogenic transcriptional factor Runx2, were also detected (Figure 3D).

**Figure 4:**
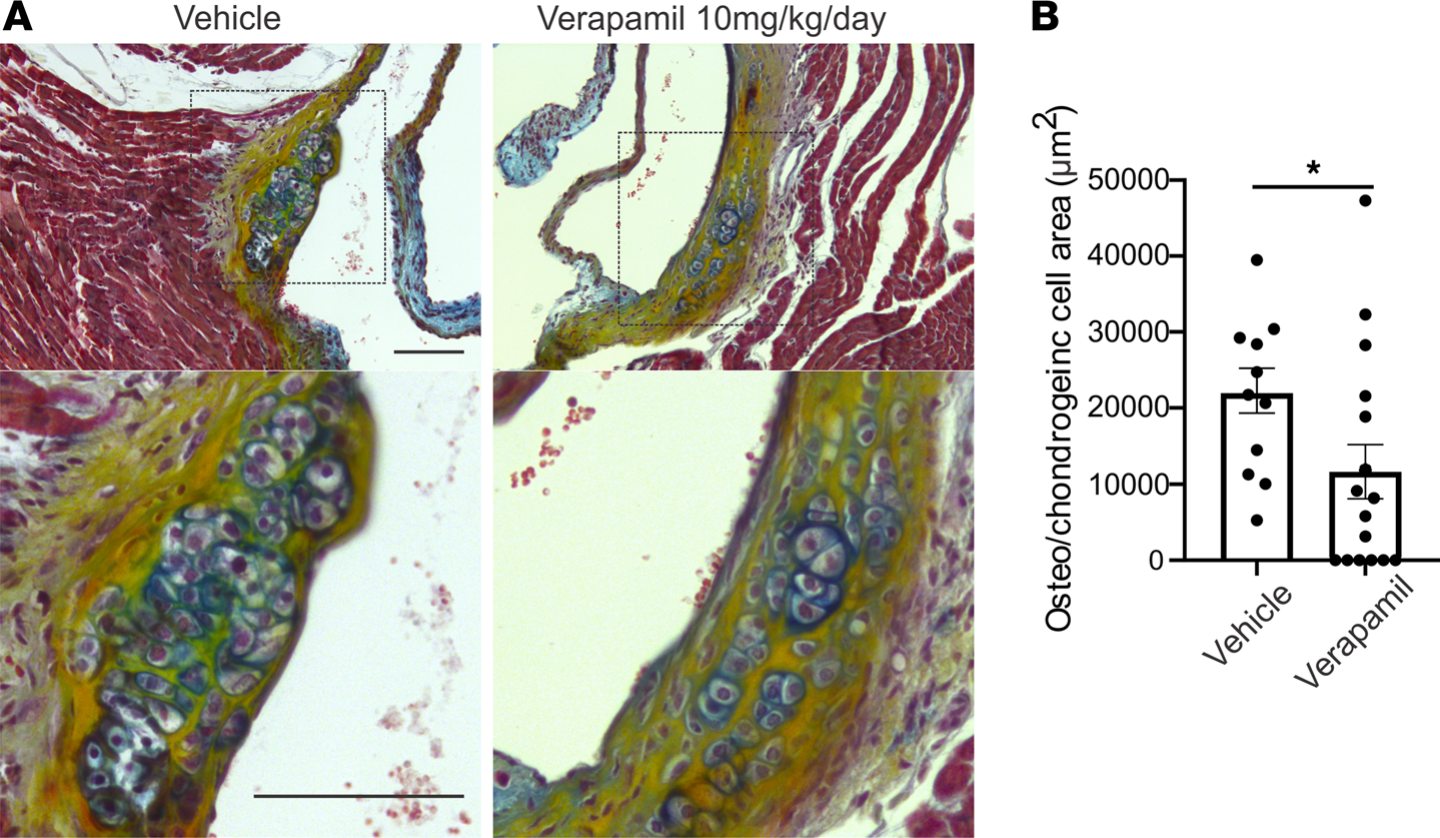
Blockade of Ca^2+^ influx through Ca_V_1.2 reduces aortic valve lesions in *Scx-Ca_V_1.2^TS^* mice. (**A**) Pentachrome stain of aortic valves from 3-month-old *Scx-Ca_V_1.2^TS^* mice. Valves were isolated after 4 weeks of treatment by mini-osmotic pumps with verapamil or vehicle control. Inset shows magnification of lesion. (**B**) Quantification of lesion area. *P = 0.04 (2-sided *t* test). Scale bars: 100 μm. Osteo, osteogenic.

Because transgenic expression of Ca_V_1.2, mimicking the increased expression of Ca_V_1.2 associated with CAVD in patients (19), led to the appearance of valve lesions and activation of the dystrophic and osteogenic signaling pathways, we next tested whether administrating a Ca^2+^ channel blocker (verapamil, 10 mg/kg/d) in vivo via a mini-osmotic pump reduced the appearance of the dystrophic lesions in the *Scx-Ca_V_1.2^TS^* model. We chose verapamil over a dihydropyridine because the *Rosa26* Ca_V_1.2^TS^ model has an engineered mutation that renders the channels insensitive to dihydropyridines (28). Mini-osmotic pumps containing verapamil or vehicle control were implanted into 2-month-old *Scx-Ca_V_1.2^TS^* mice and lesion area was quantified in valves isolated 28 days later. Lesion area was significantly reduced in verapamil-treated mice (*n* = 16; *P* < 0.05) compared with vehicle-treated control mice (Figure 4). Thus, in a model of increased Ca_V_1.2 expression in the valve, chronic blockade of Ca^2+^ influx effectively reduced valve lesions.

**Figure 5:**
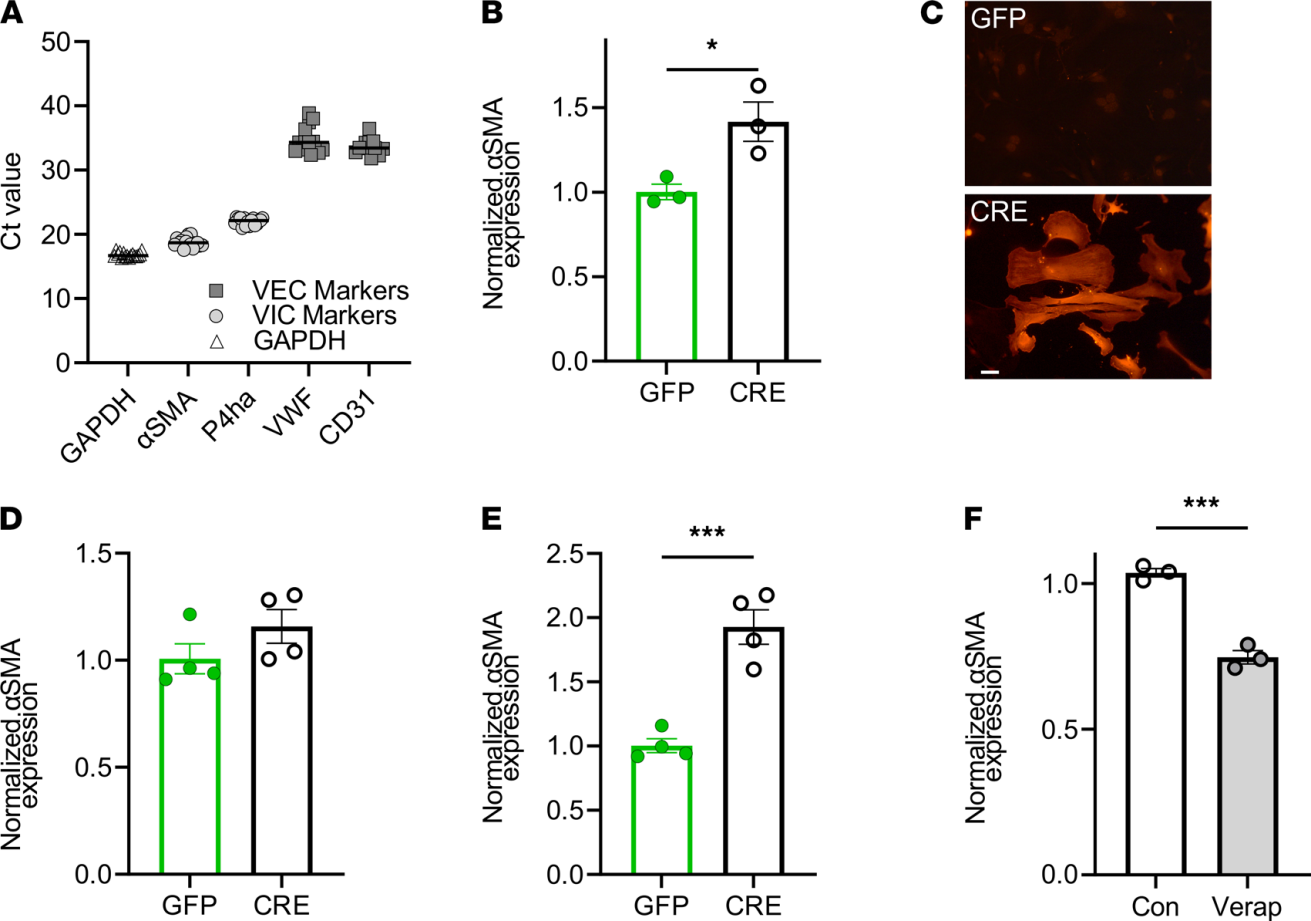
Blockade of Ca^2+^ influx through Ca_V_1.2 reduces aortic valve lesions in *Scx-Ca_V_1.2^TS^* mice. (**A**) qPCR showing expression of VIC markers (α-SMA and P4ha) compared with endothelial cell markers (VWF and CD31) in cultured VICs. (**B**) Normalized (to GAPDH) α-SMA expression after 48 hours in Ca_V_1.2^WT^-expressing VICs (after infection with virus expression GFP or Cre recombinase). *P < 0.05. (**C**) Anti–α-SMA immunofluorescence in Ca_V_1.2^TS^ VIC cultures expressing Cre or GFP. Scale bar: 50 μm. (**D**) Normalized (to GAPDH) α-SMA expression after 48 hours in WT VICs (after infection with virus expression GFP or Cre recombinase). *P* = NS. (**E**) Normalized (to GAPDH) α-SMA expression after 48 hours in Ca_V_1.2^TS^ expressing VICs (after infection with virus expression GFP or Cre recombinase). *P* < 0.001. (**F**) qPCR shows verapamil decreases relative expression of α-SMA in Ca_V_1.2^TS^ expressing VICs. ***P < 0.001. (**B**–**F**) Statistical comparisons were performed with 2-sided *t* tests. Con, control; VEC, valve endothelial cell.

To explore signaling pathways downstream of Ca^2+^ influx via Ca_V_1.2 that contributed to valve calcification, we used a mouse VIC culture system. We first confirmed that our VIC culture preparations were free of valve endothelial cell contamination by performing quantitative PCR (qPCR) for endothelial cell and VIC marker transcripts. Figure 5A shows expression levels of VIC-specific transcripts α-SMA (*Acta2*) and prolyl-4-hydrolase (*P4ha1*) that are more than 3000-fold to 60,000-fold higher than expression levels of endothelial cell markers CD31 or von Willebrand’s factor (ΔC_t_ range, 11.6–15.9).

Because the human GWAS showed a correlation between increased Ca_V_1.2 expression in the valve and valve calcification, and our mouse models demonstrated that increased Ca_V_1.2 expression in the valve is casual for valve calcification, we tested if increased Ca_V_1.2 expression in cultured VICs is sufficient to activate dystrophic signaling pathways. As a first test, we cultured VICs obtained from the mice in which *Ca_V_1.2^WT^* was inserted into the *Rosa26* locus downstream of a floxed STOP codon (see Figure 2A) and activated the *Ca_V_1.2^WT^* transgene by infecting the cells with an adenovirus expressing Cre recombinase (to remove the STOP codon) or an adenovirus expressing GFP (as a control). We then performed qPCR and immunocytochemistry for α-SMA, an indicator of myofibroblast differentiation, which showed that enhanced expression of *Cacna1c* in VICs increased α-SMA by approximately 40% 2 days after infection (Figure 5B) and led to the appearance of α-SMA stress fibers that were not present after control GFP infection (Figure 5C), thus providing initial mechanistic insight into the downstream effects of increased Ca_V_1.2 expression in VICs. We confirmed that the observed myofibroblast activation was not an artifact of Cre infection: qPCR of RNA from cultured VICs isolated from WT mice infected with GFP or Cre showed no difference in α-SMA expression levels (Figure 5D). As with the in vivo experiments, we chose verapamil over a dihydropyridine because the Ca_V_1.2^TS^ model has an engineered mutation that renders the channels insensitive to dihydropyridines (28).

Finally, we hypothesized that the additional Ca^2+^ influx through the Ca_V_1.2^TS^ mutant channel would generate an even larger increase in α-SMA expression. We infected VICs obtained from mice expressing the floxed-STOP *Ca_V_1.2^TS^* transgene knocked into *Rosa26* (Figure 2B) with either adenovirus expressing Cre recombinase (to remove the STOP codon) or GFP (control) and measured the expression of α-SMA by quantitative RT-PCR 2 days after infection. In comparison with overexpression of the Ca_V_1.2^WT^ channel and the resultant 1.4-fold increase in α-SMA (Figure 5B), expression of the gain-of-function mutant Ca_V_1.2^TS^ channel generated an approximately 2-fold increase in α-SMA expression (Figure 5E), further augmenting Ca^2+^ influx with the Ca_V_1.2^TS^ channel amplified the activation of α-SMA. Additionally, we confirmed that the resultant increase in α-SMA after expression of Ca_V_1.2 derived from Ca^2+^ influx through the transgenic Ca_V_1.2 channels, because blockade with verapamil eliminated any increase in α-SMA expression (Figure 5F).

**Figure 6:**
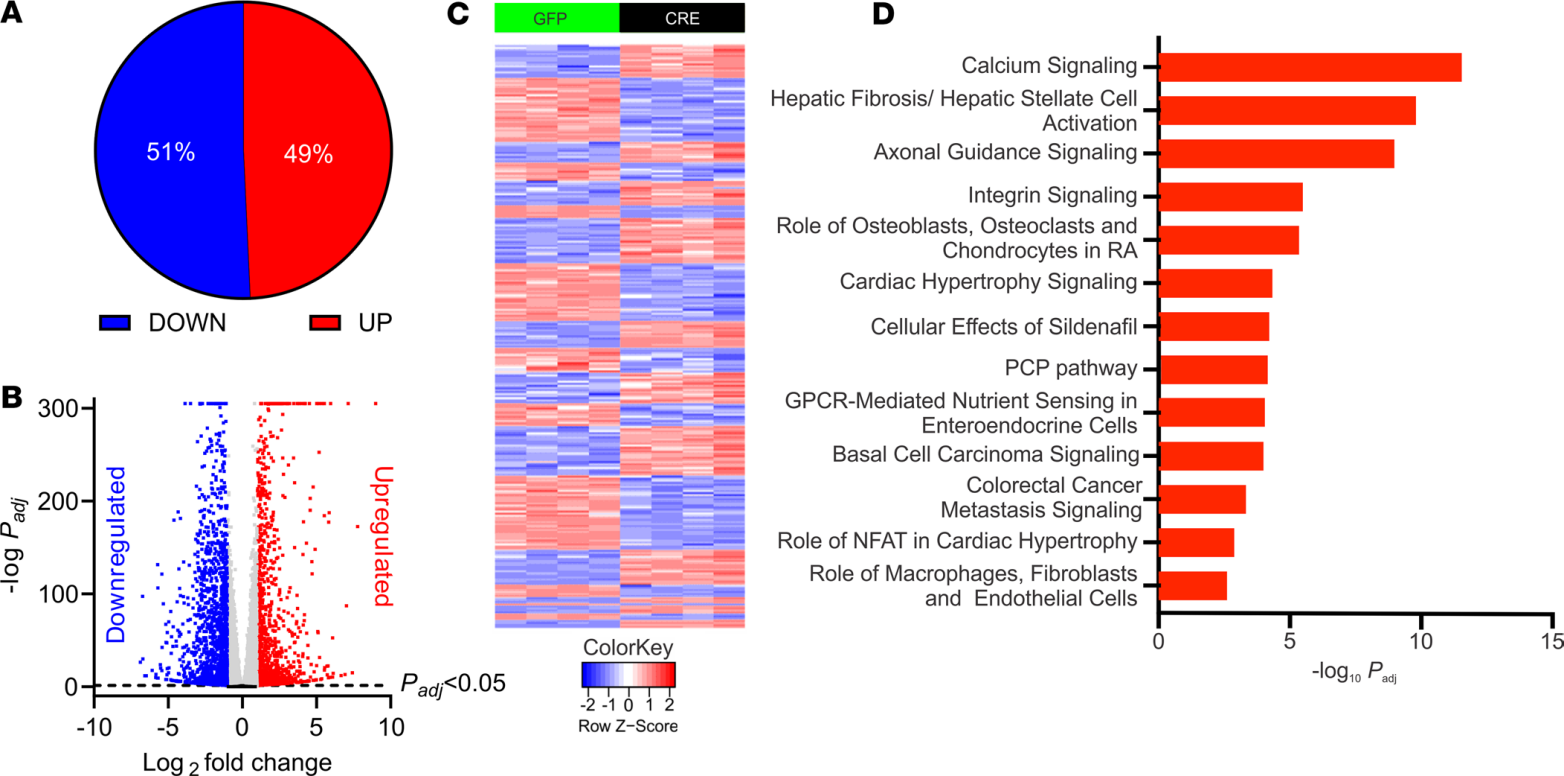
RNA-Seq of VICs infected with adenovirus expressing GFP or Cre. (**A**) Distribution of upregulated (Up) and downregulated (Down) differentially expressed genes 48 hours after viral infection. (**B**) Volcano plot of differentially expressed genes 48 hours after infection. Blue and red symbols indicate genes differentially expressed (*P_adj_* < 0.05) and log_2_-fold greater than 1.0 or less than –1.0, respectively. Gray indicates genes differentially expressed (*P_adj_* < 0.05) and log_2_-fold less than 1.0 and greater than –1.0. Black indicates genes not differentially expressed (*P_adj_* > 0.05). (**C**) Heat map of the 2724 transcripts for which expression in the Cre infected VICs was upregulated or downregulated by more than 2-fold. (**D**) Gene ontology analysis by IPA of upregulated genes. PCP, planar cell polarity; RA, rheumatoid arthritis

We next performed bulk RNA-Seq to query the initial downstream signaling pathways and factors affected by increased Ca^2+^ influx through Ca_V_1.2 in VICs. Because the gain-of-function mutant Ca_V_1.2^TS^ channel amplified the α-SMA response in cultured VICs, we exploited the Ca_V_1.2^TS^ channel for the RNA-Seq experiments. We treated cultured VICs isolated from Ca_V_1.2^TS^ mice, plated them on hard plastic with adenovirus expressing Cre recombinase or GFP (control), and isolated RNA for bulk RNA-Seq 48 hours (*n* = 4 mice each) and 72 hours (*n* = 3 mice each) after infection. Principal component analysis showed excellent separation of Cre-infected versus GFP-infected samples (Supplemental Figure 1, A and B; supplemental material available online with this article; https://doi.org/10.1172/jci.insight.155569DS1). In the 48-hour experiment, we observed upregulation of α-SMA 2.36-fold ± 0.12-fold (*n =* 4 each; adjusted *P* [*P_adj_*] < 1 × 10^–59^), similar to what we observed by qPCR (Figure 5E). Between the Cre or GFP infection samples, we observed 10,540 protein-coding genes differentially expressed (*P_adj_* < 0.05), of which 5193 were up-regulated and 5347 were downregulated (Figure 6, A and B). A heat map of the 2724 transcripts for which expression in the Cre infected VICs was upregulated or downregulated by more than 2-fold is shown in Figure 6C.

We performed a separate analysis on RNA isolated 72 hours after Cre or GFP infection (*n* = 3 each), which revealed 10,306 differentially expressed protein-coding genes, of which 5118 were upregulated and 5188 were downregulated (Supplemental Figure 1, C and D). There were 8908 differentially expressed genes common to both data sets and there was a tight correlation (*r^2^* = 0.86) between the log_2_-fold changes in expression values (Supplemental Figure 1E), suggesting limited overall differences in gene expression after the additional 24-hour exposure to increased Ca_V_1.2 expression. Therefore, to highlight the initial consequences of Ca^2+^-dependent signaling downstream of Ca_V_1.2 in the VICs, we focused our analyses on the data set obtained 48 hours after viral infection.

Gene ontology analyses by ingenuity pathway analysis (IPA) revealed that the top gene set among upregulated transcripts was the calcium-signaling pathway (*P_adj_* = 4.38 × 10^–13^) (Figure 6D), mirroring the top gene set for all SNPs that had moderate association with calcified human aortic valves (19) and consistent with upregulation of Ca^2+^ signaling by activating Ca_V_1.2^TS^ expression. A top upregulated (68.0-fold ± 16.6-fold; *P_adj_* = 2.40 × 10^–53^) Ca^2+^-signaling gene was *Nfatc2*, a transcription factor activated by the Ca^2+^-sensitive phosphatase calcineurin. The next gene set identified by IPA was the hepatic fibrosis/hepatic stellate cell–activation pathway (*P_adj_* = 5.68 × 10^–10^), also identified as the top gene set among upregulated transcripts in calcified versus normal human aortic valves (29). Processes such as increased collagen synthesis are key features of hepatic fibrosis and CAVD, and transcripts of 30 collagen genes were upregulated, 17 by more than 2-fold (Supplemental Table 1) after Ca_V_1.2 activation. Furthermore, the upregulation of collagen transcripts is another indicator of myofibroblast activation (30), as myofibroblasts lead to marked increases in collagen secretion. Moreover, IPA identified TGF-β as the top upstream regulator of the differentially expressed genes, suggesting that initial Ca^2+^ influx in mouse VICs converges on activation of a similar set of genes as application of TGF-β, and that the resulting signaling in our mouse VIC cultures more closely represents a dystrophic rather than an osteogenic process (4), consistent with our culturing of the VICs on a hard plastic surface, known to promote dystrophic calcification, rather than on material like fibronectin that better promotes osteogenic calcification (4).

One component of the hepatic fibrosis/hepatic stellate cell–activation pathway, *Agt*, was the most highly upregulated gene (511-fold ± 59-fold; *P_adj_* < 1 × 10^–300^) in our data set. *Agt* encodes angiotensinogen, the precursor of angiotensin II and a key member of the renin-angiotensin-aldosterone system (RAAS), for which individual components are tightly regulated within several feed-forward and feedback loops. The transcript level is commonly used to quantify the abundance of angiotensinogen, which is almost immediately secreted from cells (31). *AGT* is upregulated in human calcified aortic valves (29), local angiotensinogen synthesis and RAAS signaling drive aortic valve calcification (32–34), and proteomic analysis of calcified human aortic valves identified the angiotensin signaling as 1 of the upregulated pathways (35). Increased levels of the negative regulator of RAAS signaling, angiotensin-converting enzyme 2 (ACE2), is a biomarker for higher mortality rate and myocardial fibrosis in patients with CAVD (36). Therefore, we cataloged other RAAS signaling components (Supplemental Table 2), many of which are altered in CAVD, such as the *Ace2*, (Pro)renin receptor (*Atp6ap2*), and the angiotensin II receptor (*Agtr1a*), in addition to *Agt*.

While the VIC culture conditions favored dystrophic calcification over osteogenic calcification, 1 of the top signaling pathways identified by IPA analysis was the role of osteoblasts, osteoclasts, and chondrocytes in rheumatoid arthritis (Figure 6D), suggesting that Ca^2+^ influx through Ca_V_1.2 may be an activating signal for both calcification pathways. The enriched transcripts belonging to this set included multiple Wnt family members and their Frizzled receptors, as well as 3 of the 4 nuclear factor of activated T cells (*Nfatc*) genes (*Nfatc1*, *Nfatc2*, *Nfatc4*). In addition to being components of the gene set for the role of osteoblasts, osteoclasts, and chondrocytes in rheumatoid arthritis, the *Nfatc* gene products are Ca^2+^-responsive transcriptional regulators and thus serve as components of sensors capable of translating Ca^2+^ influx through Ca_V_1.2 to downstream Ca^2+^-signaling gene expression pathways (37). Together, the data from the VICs demonstrate early consequences of increased Ca^2+^ influx through Ca_V_1.2 in VICs.

## Discussion

Building upon a study showing an association between increased expression of Ca_V_1.2 in the aortic valve and CAVD (19), our combination of VIC culture and in vivo models demonstrates that increased Ca^2+^ influx through Ca_V_1.2 is, indeed, causal for the initiation of calcific lesions within the aortic valve. The location of the lesions in the hinge regions in young mice maintained on normal chow rather than a high-cholesterol diet suggests that this is a relevant model for the early aortic valve lesions described in presymptomatic individuals (10). The role of Ca_V_1.2 in this model, echoing the GWAS of patients with more advanced CAVD (19), is underlined by our experiments showing that blocking Ca_V_1.2-channel activity with verapamil reduced the α-SMA signal of myofibroblast activation in cultured VICs and decreased the valve lesions in our in vivo model. CAVD arises from myofibroblast differentiation of VICs in response to paracrine action of stimuli TGF-β, and our data show that Ca^2+^ influx through Ca_V_1.2 may be a key downstream step, because our in vivo Ca_V_1.2 mouse models showed lesion development in the absence of any such exogenous paracrine stimuli and in the absence of the hypercholesterolemia stimulus commonly used in CAVD mouse models.

The drivers for increased Ca_V_1.2 expression in diseased valves are not known, but the subsequent influx of Ca^2+^ through Ca_V_1.2 then is clearly poised to drive gene expression pathways that promote valve calcification, such as the marked upregulation of multiple collagen genes (Supplemental Table 1) or the activation of local RAAS signaling (Supplemental Table 2) that is known to promote valve calcification and may also be a promising target, based on a retrospective analysis (38).

That verapamil was effective in cultured VICs suggests the ability of Ca_V_1.2 Ca^2+^ channel antagonists to reduce myofibroblast activation is independent of an effect on blood pressure. While these agents may not be recommended for individuals with severely advanced disease (39), in part because Ca^2+^ channel antagonists and other agents that reduce afterload are generally contraindicated in severe aortic valve disease, Ca^2+^ channel antagonists could be well tolerated earlier in the disease process, such as when aortic sclerosis is recognized by echocardiography. The similarity between the location and morphology of these lesions and those found in presymptomatic individuals (10) underscores the translational potential of Ca^2+^ channel antagonists for early CAVD. Although dihydropyridines, which have less negative inotropy, may be the Ca^2+^ channel antagonist of choice, we were unable to assess them in our transgenic models, because of a designed dihydropyridine-resistance mutation to allow pharmacological distinction of the transgenic Ca_V_1.2 channels from endogenous channels (28). Moreover, because critical steps leading to CAVD, such as dystrophic calcification and osteogenesis, mimic processes identified in atherosclerotic calcification (40, 41), the efficacy of a Ca^2+^ channel antagonist in the VIC cultures resonates with previous clinical trial data (e.g., PREVENT, CAMELOT, ACCOMPLISH) showing that Ca_V_1.2 Ca^2+^ channel antagonists reduced cardiovascular events and caused regression of atherosclerotic plaques (42–44), which is especially notable because the CAMELOT trial researchers observed a benefit with amlodipine in patients with normal blood pressure. Despite the parallels between CAVD and atherosclerosis, pharmacological treatments that have been successful for atherosclerosis failed for CAVD (e.g., lipid lowering with statins) (45–48), emphasizing the need for the identification of new targets and the development of novel therapies for CAVD by uncovering disease mechanisms. Ca_V_1.2 Ca^2+^ channel antagonists may offer 1 such therapy that, to our knowledge, has not yet been investigated.

The RNA-Seq data obtained from cultured VICs provide several clues to important mediators downstream of Ca_V_1.2 and suggestions for additional therapeutic interventions and disease mechanisms. For example, Supplemental Table 3 lists key upregulated, Ca^2+^-sensitive signaling genes, including those encoding Ca^2+^/calmodulin kinases (*Camk2a* and *Camkk1*), the NFATc components of the Ca^2+^-sensitive phosphatase calcineurin complex that mediate Ca^2+^-dependent transcription (*Nfatc1*, *Nfatc2*, *Nfatc4*), and the Ca^2+^-binding second messenger and transcriptional regulator calreticulin (*Calr*), with *Nfatc2* being the top upregulated transcript. Thus, targeting NFAT-dependent transcription with inhibitors such as cyclosporine or tacrolimus may offer opportunities to prevent or delay progression of CAVD beyond the suggested efficacy of Ca_V_1.2 Ca^2+^ channel antagonists such as verapamil that was effective in our mouse model. Antagonism of the calcineurin-signaling pathway prevents graft atherosclerosis (49), suggesting an additional parallel between mechanisms driving atherosclerosis and CAVD.

Another example of a gene upregulated by Ca^2+^ influx through Ca_V_1.2 that provides potential mechanistic insight is *Ttr* (16.47-fold ± 7.73-fold; *P_adj_* = 3.05 × 10^–6^). *Ttr* encodes transthyretin, a protein that deposits in the heart and cause cardiac amyloidosis. There are 2 types of cardiac amyloidosis, hereditary transthyretin cardiac amyloidosis that results from mutations in TTR, and senile transthyretin cardiac amyloidosis, in which WT transthyretin deposits in the myocardium and valves. Deposition of transthyretin is increasingly recognized in valves excised from patients undergoing valve replacement for CAVD (50), and cardiac amyloidosis is increasingly recognized as a comorbidity of CAVD (51). How WT transthyretin leads to amyloid deposition in senile cardiac amyloidosis is unclear, but a transgenic mouse model with *TTR* overexpression demonstrated amyloid deposition (52). Thus, the marked upregulation of *Ttr* in the VICs may provide a mechanism.

The mechanisms by which Ca_V_1.2, a voltage-gated Ca^2+^ channel that is usually considered in the context of excitable cells such as neurons or cardiomyocytes, is regulated in fibroblast-like VICs is not clear; membrane voltage in VICs has not been actively studied. Nevertheless, there is growing evidence that Ca_V_1.2 confers important functions in nonexcitable cells. Chondrocytes and osteoblasts appear to be prime examples, as others and we have shown (21–23, 27). Moreover, individuals with Timothy syndrome with the Ca_V_1.2 G406R gain-of-function mutation suffer from various phenotypes in multiple nonexcitable tissues (26, 53), although these individuals have not been reported to have CAVD, likely because most known patients have short life spans. For the aortic valve, which is subject to mechanical shear stress, 1 possible means of further activating Ca_V_1.2 in vivo may be via activation of mechanosensitive channels such as Piezo1 that have been found in bone. Osteocytes express high levels of *Piezo1* in static conditions and the *Piezo1* transcripts increase with shear stress (54, 55). Stretch and mechanical forces cause Piezo1 or its homolog Piezo2 to open, providing an inward depolarizing current, including Ca^2+^ (56). Shear stress is a potent driver of the CAVD pathway (57) and, in our VIC cultures, *Piezo1* is highly expressed: it is in the top 0.02% of all protein coding transcripts by read abundance.

In conclusion, our RNA-Seq data from VICs in which increased Ca^2+^ influx through Ca_V_1.2 identified multiple differentially expressed genes and signaling pathways common in CAVD, show that the association between *CACNA1C* SNPs and increased Ca^2+^ influx through Ca_V_1.2 with CAVD is, indeed, causal. Moreover, these collected data provide mechanistic insight. Our in vivo mouse-model studies showed increased Ca^2+^ influx through Ca_V_1.2 in the aortic valve resulted in lesion formation with dystrophic, chondrogenic, and osteogenic characteristics, reflecting later signaling events, which we hypothesize are driven by the initial cascade of gene expression changes observed in the cultures. An important result from these studies is that, because Ca_V_1.2 is druggable, targeted treatment with commonly used Ca^2+^ channel antagonists, if applied early in the disease process, may offer a therapeutic strategy, as we showed with verapamil. Our RNA-Seq data sets and these newly developed models suggest that further definition of the signaling events from Ca^2+^ influx through Ca_V_1.2 to myofibroblast activation and resultant valve calcification may reveal additional therapeutic targets for development.

## Methods

### Human aortic valve immunofluorescence.

Immunofluorescent staining of human tissue samples was performed as previously described (58). Briefly, OCT-embedded (Tissue-Tek) frozen sections (7 μm) from control valves (obtained for heart transplant surgery) or calcified valves removed during surgical aortic valve replacement were fixed in an acetone/methanol solution for 10 minutes at 4°C. After blocking for 1 hour, the sections were incubated with a custom-made polyclonal anti-α_1C_ (Ca_V_1.2) antibody (Yenzym, 1:50 dilution) (59–61) and a monoclonal anti-Runx2 antibody (Abnova, H00000860-M05).

### RNA-Seq and bioinformatics.

For the 48-hour data sets (*n* = 4 mice each) and the 72-hour data sets (*n* = 3 mice each), library preparation and sequencing were performed at Weill Cornell Medicine Genomic Core Facility. The sequencing libraries were sequenced with single-end 50 bps on a HiSeq 4000 sequencer (Illumina). The raw sequencing reads in binary base call format were processed through bcl2fastq 2.19 (Illumina) for FASTQ conversion and demultiplexing. RNA reads were aligned and mapped to the GRCm38 mouse reference genome by STAR (version 2.5.2; https://github.com/alexdobin/STAR) (62), and transcriptome reconstruction was performed by Cufflinks (version 2.1.1; http://cole-trapnell-lab.github.io/cufflinks/). The abundance of transcripts was measured with Cufflinks in fragments per kilobase of exon model per million mapped reads (63, 64). Gene expression profiles were constructed for differential expression, cluster, and principle component analyses with the DESeq2 package (https://bioconductor.org/packages/release/bioc/html/DESeq2.html) (65), eliminating genes with insufficient base mean reads. For the canonical pathway and gene network analysis, data were analyzed via IPA (QIAGEN). Raw data are deposited in the Gene Expression Omnibus (GEO GSE151520).

### qPCR.

RNA was isolated from cultured mouse VICs using the RNeasy Mini Kit (QIAGEN) according to the manufacturer’s instructions. Reverse transcription was performed using iScript cDNA Synthesis Kit (Bio-Rad). qPCR was performed in duplicate for each sample with QuantStudio 3 (Applied Biosystems) using SYBR green-based detection chemistries (Bio-Rad). Relative expression was quantified using the Ct method, except in Figure 5A, in which the raw Ct values are reported to emphasize the almost complete lack of valvular endothelial cell markers. GAPDH was used for a reference gene. For primer sequences used for qPCR, see Supplemental Table 4 (66–68).

### Animals.

C57BL/6J mice (B6) (catalog 00064) and Ca_V_1.2^lacz/+^ reporter mice (catalog 0005783) were purchased from Jackson Laboratory. *ScxCre* mouse (69) was provided by Ronen Schweitzer (Oregon Health and Science University, Portland, Oregon, USA). STOP-floxed WT or a G406R mutant *Ca_V_1.2WT*^/+^ and *Ca_V_1.2^TS/+^* mice were described previously (28). Data from male and female mice were analyzed after initial observations did not reveal a sex difference.

### Aortic VIC isolation and in vitro assays.

Mouse primary aortic VICs were isolated as described previously (70). Briefly, aortic valves were dissected and pooled together (*n* = 3–5) and digested with collagenase to eliminate valve endothelial cells (VECs) and isolate VICs. Cells from passages 3–8 were used. Adenovirus expressing Cre recombinase (Vector Biolabs, 1045) or GFP (Vector Biolabs, 1060) were used to infect cells. For the verapamil assay, 10 μM verapamil was added to Ca_V_1.2^TS^ cells in addition to adenovirus expressing either Cre recombinase or GFP. After 2 days of incubation, RNA was isolated and qPCR was performed.

### Histology, pentachrome staining, and immunofluorescent staining.

*ScxCre;Ca_V_1.2^TS/+^* and *ScxCre;Ca_V_1.2^WT/+^* mice and their control littermates (*Ca_V_1.2^TS/+^* and *Ca_V_1.2^WT/+^*) were sacrificed. Hearts were removed and fixed in 4% paraformaldehyde for 1 hour at room temperature. Tissue was further dehydrated through ethanol gradient washes, embedded in paraffin, and sectioned at 10 μm. Movat’s pentachrome staining was performed according to the manufacturer’s protocol (American MasterTech Scientific). For Aggrecan (1:500; Millipore, AB1031) and Sox9 (1:500; Millipore, AB5535) staining, tissues were treated with chondroitinase (200 mU/mL; Sigma) before blocking. For Runx2 (1:100; Novus Biologicals, NBP1-77472) and α-SMA (1:200; Abcam, ab5694), antigen retrieval was performed using sodium citrate (10 mM, pH 6) before blocking.

### Determination of calcium deposition in aortic valve.

Hearts from *ScxCre;Ca_V_1.2^TS/+^* and *ScxCre;Ca_V_1.2^WT/+^* mice and their control littermates (*Ca_V_1.2^TS/+^* and *Ca_V_1.2^WT/+^*) were fixed in 4% paraformaldehyde, embedded in paraffin, and sectioned at 10 μm. For von Kossa staining to detect calcium deposition in the aortic valve (71), sections were incubated in 1% aqueous silver nitrate solution for 20 minutes in the UV chamber (Fisher Scientific, FB-UVXL-1000). Slides were rinsed in water, excess silver was removed with 5% sodium thiosulfate for 5 minutes, and counterstained with nuclear fast red. For alizarin red staining, sections were fixed in 4% paraformaldehyde and incubated in alizarin red solution for 30 minutes.

### Mini-osmotic pump implantation.

A mini-osmotic pump (Alzet, 2004) filled with either vehicle (DMSO diluted 1:1 in water) or verapamil solution (10 mg/kg/d) was implanted s.c. for infusion at a rate of 10 mg/kg/d. Incision was closed with a surgical stapler. After 28 days of drug delivery through a mini osmotic pump, mice were sacrificed and the heart samples were processed for histology.

### Statistics.

Data analysis was performed using GraphPad Prism, version 8.4. All averaged data are presented as mean ± SEM. Statistical significance was determined using 2-tailed *t* tests, a 2-way ANOVA with post hoc correction for multiple comparisons, or χ^2^ test. *P* < 0.05 was considered statistically significant. For RNA-Seq differential expression analyses, pairwise comparisons between 2 or more groups were made using parametric tests in which read counts follow a negative binomial distribution with a gene-specific dispersion parameter. *P_adj_* values were calculated on the basis of the Benjamin-Hochberg method to adjust for multiple testing.

### Study approval.

Animals were handled according to NIH Guide for the Care and Use of Laboratory Animals. The research was approved by the Weill Cornell Research Animal Resource Center.

Human aortic valve samples were obtained from the valve replacement and heart transplantation surgeries at Mount Sinai Hospital, New York, New York. The study was approved (GCO 13-1821/HS 13-00741) by the IRB of the Icahn School of Medicine at Mount Sinai. All patients gave written informed consent prior to surgery.

## Author contributions

MM, RB, SOM, and GSP designed the research studies. MM and RB conducted experiments, acquired data, analyzed data. MS, YH, and AG conducted experiments, acquired data, and analyzed data. MM, SOM, and GSP wrote the manuscript.

## Supplementary Material

Supplemental data

## Version 1 02/01/2022

In-Press Preview

## Version 2 03/08/2022

Electronic publication
