# Supplementary material for: Increased Ca2+ influx through CaV1.2 drives aortic valve calcification
Source: JCI Insight. 2022 Mar 8;7(5):e155569. doi: 10.1172/jci.insight.155569 (PMC8983132; doi:10.1172/jci.insight.155569)
Supplement: Supplemental data [file jciinsight-7-155569-s191.pdf]

SUPPLEMENTAL DATA FOR

Increased Ca^{2+} influx through $Ca_V1.2$ drives aortic valve calcification

Maiko Matsui[1], Rihab Bouchareb[2], Mara Storto[1], Yasin Hussain[1], Andrew Gregg[1], Steven O. Marx[3] and Geoffrey S. Pitt[1,2]

From the [1]Cardiovascular Research Institute, Weill Cornell Medicine, New York, NY; [2]Zena and Michael A. Wiener Cardiovascular Institute, Icahn School of Medicine at Mount Sinai, New York, NY; and [3]Division of Cardiology, Department of Medicine, and Department of Pharmacology, Vagelos College of Physicians and Surgeons, Columbia University, New York, NY

Supplemental Table S1. Collagen genes up-regulated more than two-fold after infection with adenovirus expressing *Cre* recombinase.

Gene	**Log_2Fold Change**	**P_{adj}**
Col1a1	1.18±0.10	4.40E-247
Col4a4	1.93±0.05	5.40E-11
Col5a2	2.72±0.04	3.37E-166
Col5a3	1.01±0.03	5.24E-120
Col6a3	2.29±0.14	5.27E-247
Col7a1	2.16±1.33	1.80E-49
Col8a2	2.26±0.08	1.37E-21
Col9a3	2.56±0.14	1.01E-34
Col11a2	2.11±0.20	1.17E-110
Col12a1	1.36±0.05	<1.0E-300
Col13a1	1.96±0.08	1.09E-15
Col16a1	2.58±0.08	3.16E-149
Col18a1	2.30±0.06	<1.0E-300
Col20a1	3.71±0.24	2.42E-74
Col26a1	1.42±0.17	1.77E-03
Col27a1	1.18±0.10	<1.0E-300
Col28a1	1.26±0.04	1.84E-24

Supplemental Table S2. Expression of altered RAAS signaling components after infection with adenovirus expressing *Cre* recombinase.

Gene	Log_2Fold Change	P_{adj}
Agt	8.99±0.17	<1.0E-300
Ace2	4.32±0.16	2.3E-155
Atp6ap2	2.05±0.04	<1.0E-300
Agtrap	0.46±0.06	1.1E-11
Renbp	0.33±0.05	7.74E-11
Agtr1a	-2.3±0.22	1.19E-26
Agtr1b	-2.5±0.85	9.42E-05

Supplemental Table S3. Ca^{2+} sensitive genes (from: http://software.broadinstitute.org/gsea/msigdb/cards/KEGG_CALCIUM_SIGNALING_PATHWAY).

Gene	Log_2Fold Change	P_{adj}
Nfatc2	6.08±0.40	2.28E-54
Atp2a3	5.52±0.19	7.19E-187
P2rx1	4.96±1.11	1.70E-08
Atp2a1	4.84±0.62	3.05E-17
Grpr	4.43±0.38	1.83E-32
Mylk	3.52±0.11	1.86E-222
Cacna1s	3.09±1.25	8.11E-05
P2rx5	3.02±0.14	4.58E-98
Gna14	2.91±0.84	8.81E-06
Rcan1	2.73±0.07	2.48E-296
Adcy4	2.42±0.34	1.56E-13
Gna15	2.40±0.24	2.56E-24
Tnnc2	2.19±1.43	1.21E-03
Ptger1	2.13±0.16	2.70E-41
Cacna1c	2.09±0.03	0.00E+00
P2rx3	2.05±0.12	1.35E-62
Camk2a	2.05±0.11	3.81E-67
Nos2	1.99±1.37	1.87E-03
Nfatc1	1.85±0.05	3.85E-211
Rcan2	1.80±0.09	1.02E-89
Bst1	1.75±0.24	2.21E-14
Mylk2	1.65±0.64	2.09E-04
Cacna1g	1.47±0.04	1.39E-209
Camkk1	1.45±0.08	1.13E-70
Tbxa2r	1.42±0.13	7.06E-29
P2rx6	1.40±0.17	5.11E-17
Cacna1b	1.38±0.40	1.39E-05
Prkcg	1.37±0.19	8.86E-15
Adrb2	1.32±0.15	2.16E-19
Calr	1.20±0.04	1.65E-160
Phkg1	1.16±0.22	5.91E-09
Gna11	1.16±0.03	3.24E-223
Nfatc4	1.11±0.03	5.71E-187
Atp2b3	1.07±1.09	1.94E-02
Adra1b	-1.2±0.18	3.14E-14
Mylk3	-1.3±0.36	2.04E-06
Adora2b	-1.4±0.11	7.25E-41
Adrb1	-1.4±0.47	3.54E-05
Oxtr	-1.7±0.75	4.00E-04
Htr2a	-1.9±0.10	5.80E-82
Adcy8	-1.9±0.18	7.57E-29
Plcb1	-2.1±0.13	3.83E-61
Grin2a	-2.2±1.28	7.91E-04
Htr2b	-2.4±0.43	1.50E-09
Adora2a	-2.6±0.27	5.93E-22

Supplemental Table S4. Primers used for quantification of mRNA expression by qRT-PCR.

**Gene name**	**Forward primer (5′ 🕒 3′)**	**Reverse primer (5′ 🕒 3′)**	**Primerbank ID (66, 67)**
Asma (acta2)	GTCCCAGACATCAGGGAGTAA	TCGGATACTTCAGCGTCAGGA	-
Runx2	TTCAACGATCTGAGATTTGTGGG	GGATGAGGAATGCGCCCTA	-
Vwf	CTTCTGTACGCCTCAGCTATG	GCCGTTGTAATTCCCACACAAG	33186908a1
Pecam1 (cd31)	ACGCTGGTGCTCTATGCAAG	TCAGTTGCTGCCCATTCATCA	26332198a1
P4ha1	AGCCACCATTTCAAACCCAGT	GCCAAGCACTTTTGCTAATTCTG	33859596a1
Cacna1c	GCTGACGGTGTTCCAGTGTA	CCAGGTAGCCTTTGAGATCTTCT	-
Gapdh	TGTCAGCAATGCATCCTGCA	CCGTTCAGCTCTGGGATGAC	-

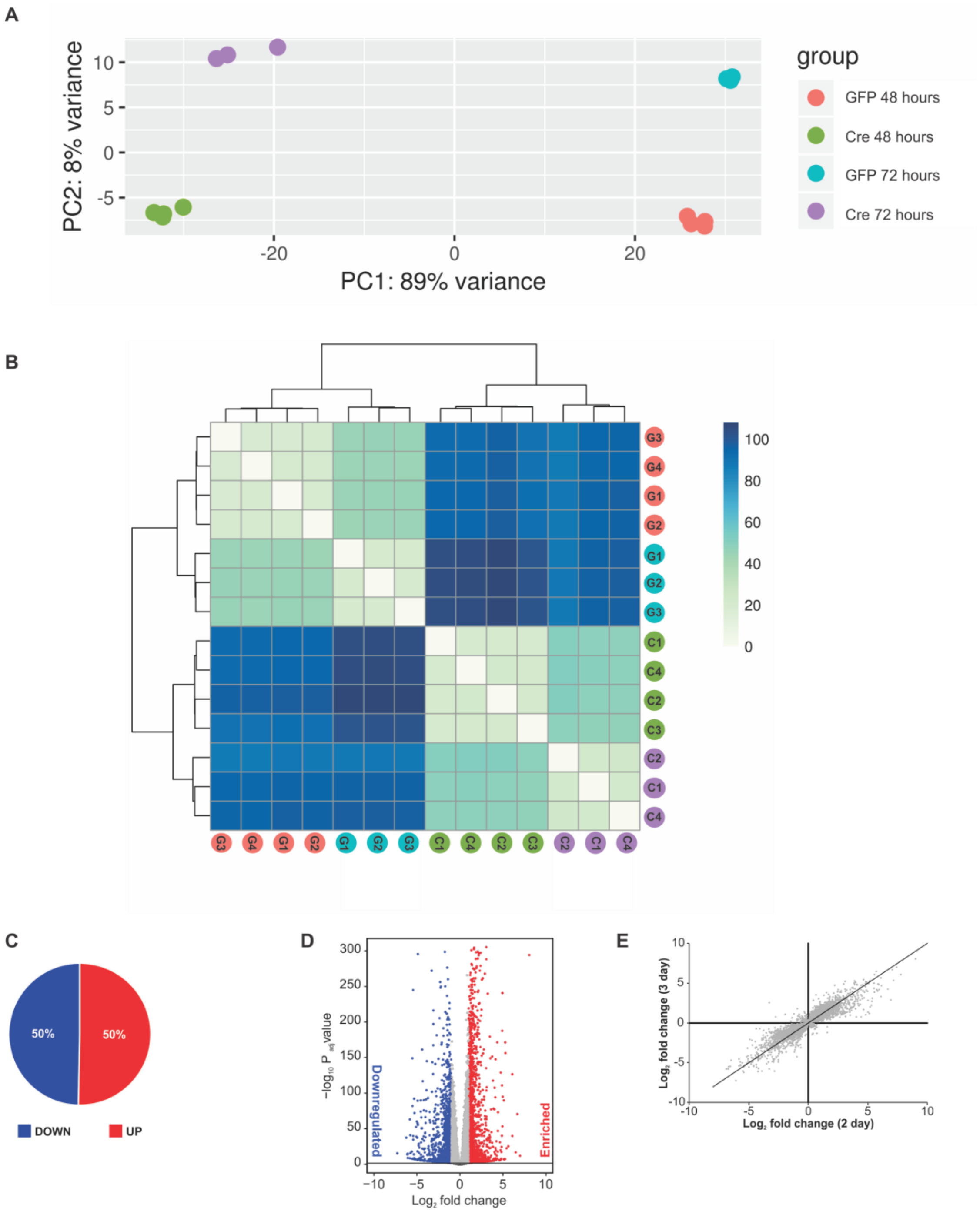

Figure S1: RNA-Seq of cultured valve interstitial cells. ***A***, PCA of RNA transcripts isolated from VICs infected with adenovirus expressing Cre recombinase or GFP either 48h (*N*=4 each) or 72h (*N*=3 each) post infection. ***B***, Heatmap of sample-to-sample distances. The analysis separates groups by the treatment (GFP ["G"] or Cre ["C"]) and the duration of treatment (48h or 72h). ***C***, Distribution of up-regulated and down-regulated differentially expressed genes 72h after viral infection. ***D***, Volcano plot of differentially expressed genes 72 h after infection. Gray indicates genes differentially expressed (P_{adj}<0.05) and Log$_2$fold <1.0 or >1.0. ***E***, Comparison of Log$_2$fold changes in 48h (2 day) and 72 h (3 day) datasets.
